# Endovascular treatment of vertebral and basilar artery aneurysms with low-profile visualized intraluminal support device

**DOI:** 10.1186/s12883-021-02180-1

**Published:** 2021-05-15

**Authors:** Quanlong Hong, Wenqiang Li, Jing Ma, Peng Jiang, Yisen Zhang

**Affiliations:** 1 Department of Neurology, The First Hospital of Quanzhou Affiliated to Fujian Medical University, Quanzhou, China; 2 Department of Interventional Neuroradiology, Beijing Neurosurgical Institute and Beijing Tiantan Hospital, Capital Medical University, NansanhuanXilu 119, Fengtai District, Beijing, 100070 China; 3 Department of Neurosurgery, The First Affiliated Hospital of Zhengzhou University, Zhengzhou, Henan China; 4 Department of Echocardiography, Shanghai Xuhui Central Hospital, Zhongshan-Xuhui Hospital, Fudan University, Shanghai, China

**Keywords:** Vertebral and basilar artery aneurysms_1_, Low-profile visualized intraluminal support device _2_, stent_3_, Endovascular treatment_4_, complications_5_

## Abstract

**Background:**

The Low-profile Visualized Intraluminal Support (LVIS) device is a self-expanding, nitinol, single-braid, closed-cell device that was recently developed for endovascular embolization of intracranial aneurysms. However, current knowledge regarding the use of LVIS devices to treat vertebral and basilar artery aneurysms is limited. We aimed to evaluate the feasibility, efficacy, and safety of the LVIS device for treating vertebral and basilar artery aneurysms.

**Methods:**

Between January 2015 and December 2017, patients with vertebral and basilar artery aneurysms treated using LVIS stents were enrolled in this study. We analyzed patients’ demographic, clinical and aneurysmal characteristics, procedural details, complications, and angiographic and clinical follow-up results.

**Results:**

We identified 63 patients with 64 vertebral and basilar artery aneurysms who underwent treatment with (*n* = 59) or without (*n* = 5) LVIS stenting, including 10 patients with ruptured aneurysms. Forty-one aneurysms were located at the vertebral artery, and 23 at the basilar artery. Intraprocedural-related complications developed in three (4.8%) patients, while none of these patients developed morbidities or died during follow-up. Three patients developed post-procedural complications (4.8%). Two patients experienced ischemic events immediately post-procedure. A minor permanent morbidity developed in one of the two patients (1.6%). The mortality rate was 1.6%, for that the patient died of brainstem hemorrhage after 1 month of follow-up. At a mean follow-up of 12.5 months, 39/43 (90.7%) patients had stable or improved aneurysms, and four (9.3%) had recanalized.

**Conclusions:**

LVIS device of vertebral and basilar artery aneurysms may be an acceptable safety profile and may represent a reasonable treatment option in the short-term. Long-term and larger cohort studies are necessary to validate our results.

**Supplementary Information:**

The online version contains supplementary material available at 10.1186/s12883-021-02180-1.

## Background

Vertebral and basilar artery aneurysms with a poor natural history can cause subarachnoid hemorrhage and posterior circulation ischemia, resulting in high morbidity and mortality [1–3]. Treating vertebral and basilar artery aneurysms by clipping is usually challenging; therefore, endovascular coil embolization has been widely used for these aneurysms [4]. However, coiling alone to treat complex vertebral and basilar artery aneurysms remains controversial because of the poor long-term stability. To improve the efficacy of endovascular treatment, intracranial stent was introduced to reduce recurrence rates by the mechanical barrier, flow diversion, and biological effects [5, 6]. The Low-profile Visualized Intraluminal Support device (LVIS®; MicroVention-Terumo, Tustin, CA) is a novel, self-expanding, braided stent with a closed-cell construction made of nitinol, which could provide a higher degree of metal coverage (approximately 23%) than conventional laser-cut stents such as the Enterprise (Codman Neurovascular, Miami Lake, FL), and Solitaire (Covidien, Irvine, CA) stents, but a lower degree of metal coverage than with flow diverters [7]. The LVIS design characteristics could improve the durability of endovascular treatment while avoiding impact on side branches [8]. However, the safety and efficacy of the LVIS stent to treat vertebral and basilar artery aneurysm requires further investigation. Therefore, we performed this study to evaluate the safety and efficacy of endovascular treatment for vertebral and basilar artery aneurysms using the LVIS stent.

## Methods

This retrospective study was approved by the Institutional Review Board of Beijing Tiantan hospital. Verbal informed consent was obtained from the patients or their family members during hospitalization.

### Patient selection

From January 2015 to December 2017, a total of 63 patients with 64 vertebral and basilar artery aneurysms treated with the LVIS stent were enrolled in this consecutive study. We recorded patients’ clinical and aneurysmal characteristics, namely age, sex; cigarette smoking; alcohol intake; diabetes mellitus; hyperlipidemia; hypertension; aneurysm length, diameter, and maximum size; complications; follow-up interval; and angiographic and clinical follow-up results.

### Endovascular procedures

Before the procedure, patients with unruptured vertebral and basilar artery aneurysms were premedicated with a dual-antiplatelet regimen (75 mg of clopidogrel and 100 mg of aspirin daily) for at least 3 days. For patients with ruptured vertebral and basilar artery aneurysms, we administered a loading dose of antiplatelet medication (300 mg aspirin and 300 mg clopidogrel) orally or through a stomach tube 4 h before the procedure. The endovascular procedures were then performed under general anesthesia with systemic heparin administration. Using the native and road map images, we deployed the LVIS stent to reconstruct the parent artery. If the vertebral and basilar artery aneurysmal sac contained sufficient space for coils, we performed coil embolization with the jailed-catheter technique. Treatment with the stent alone was preferred if the aneurysmal sac was not suitable for coil embolization. In accordance with the immediate angiographic findings just after deploying the first stent, subsequent overlapping stents were considered if a single stent was insufficient to remodel the blood flow [9]. After the procedure, dual antiplatelet agents (75 mg clopidogrel and 100 mg aspirin) were provided orally once daily for 6 weeks, and 100 mg aspirin was continued for the next 6 months.

### Clinical and angiographic follow-up

A 6-month angiographic follow-up was recommended, and follow-up magnetic resonance angiography or computed tomography angiography was performed annually. Any aneurysm showing an increased percentage of contrast filling of the aneurysmal sac on follow-up angiography compared with the control angiogram was considered recurrence, and other aneurysms were regarded as stable or improved. The modified Rankin Scale (mRS) score was used to measure patients’ clinical outcomes at follow-up visits or by telephone interview. We categorized patients’ results as favorable (mRS, 0–2) or unfavorable (mRS, 3–6) in accordance with the last clinical follow-up findings.

## Results

### Patients’ clinical and aneurysmal characteristics

Patients’ baseline and aneurysmal characteristics are shown in Tables 1 and 2. Sixty-three consecutive patients with 64 vertebral and basilar artery aneurysms were included in the study, constituting 19 women and 44 men, ranging in age from 18 to 71 years (mean, 52.2 years). Twenty-five patients (39.7%) presented with chronic headache/dizziness, 17 patients (27.0%) with neurological deficit, 11 (17.5%) aneurysms were incidental, and 10 (15.9%) patients had subarachnoid hemorrhage. Patient comorbidities were smoking (*n* = 24, 38.1%), diabetes mellitus (*n* = 5, 7.9%), hyperlipidemia (*n* = 6, 9.5%), and hypertension (*n* = 33, 52.4%). Fifty-seven aneurysms (89.1%) were dissecting aneurysms, and 7 (10.9%) were saccular aneurysms. Forty-one aneurysms (64.1%) were located at the vertebral artery, and 23 (35.9%) were located at the basilar artery. The mean length of the aneurysms was 11.1 ± 7.4 mm, and mean aneurysmal diameter was 7.9 ± 4.3 mm. There were 3 tiny aneurysms (< 3 mm), 30 small aneurysms (3–10 mm), 26 large aneurysms (10–25 mm), and 5 giant aneurysms (≥ 25 mm).

**Table 1:** Baseline information of the patients (*n* = 63) with vertebral and basilar artery aneurysms treated with stent-assisted coil embolization using LVIS device

Characteristics	No. (%)
Mean age (years) (mean ± SD)	52.2 ± 10.9
Gender	
Women	19 (30.2)
Men	44(69.8)
Main symptoms	
Chronic Headache/dizziness	25 (39.7)
Neurological deficits	17 (27.0)
Incidental	11 (17.5)
Acute SAH	10 (15.9)
Risk factors	
Smoking	24 (38.1)
DM	5 (7.9)
Hyperlipidemia	6 (9.5)
HBP	33 (52.4)
Pre-operative mRS scale (*n* = 63)	
0	27 (42.9)
1	23 (36.5)
2	3 (4.8)
3	7 (11.1)
4	3 (4.8)
Clinical follow-up available	62 (98.4)
mRS scale at lasted follow up (*n* = 42)	
0	38 (61.3)
1	21 (33.9)
2	0 (0.0)
3	1 (1.6)
4	1 (1.6)
5	0 (0.0)
6	1 (1.6)^a^
Follow-up period (months) (mean ± SD (range))	24.3 ± 10.6 (12–38)

*SAH* Subarachnoid hemorrhage, *HBP* High blood pressure, *DM* Diabetes mellitus, *SD* Standard deviation, *mRS* Modified Rankin Scale

^a^One patient died of brain stem failure caused by recurrence of a vertebral dissecting aneurysm

**Table 2:** The information of vertebrobasilar dissecting aneurysms, procedural details and outcomes (*n* = 64)^a^

Characteristics	No. (%)
Aneurysm Type	
Saccular	7 (10.9)
Dissecting	57 (89.1)
Ruptured	
Yes	10 (15.6)
No	54 (84.4)
Aneurysm location	
Vertebral artery	41 (64.1)
Basilar artery	23 (35.9)
Mean aneurysm length (mm) (mean ± SD)	11.1 ± 7.4
Mean aneurysm diameter (mm) (mean ± SD)	7.9 ± 4.3
Aneurysm size (mm)	
Tiny (< 3)	3 (4.7)
Small (≥3, < 10)	30 (46.9)
Large (≥10, < 25)	26(40.6)
Giant (≥25)	5 (7.8)
Procedure	
LVIS stent only (n (%))	5 (7.8)
LVIS stent and coiling (n (%))	59 (92.2)
No of stents (n (%))	
Single LVIS	48 (75.0)
Multiple LVIS	16 (25.0)
In-stent stenosis (n (%))	2 (3.2%)
Raymond Scale (*n* = 64)	
1	32 (50.0)
2	23 (35.9)
3	9 (14.1)
Angiographic follow-up available	43 (67.2)
Follow-up duration (months) (mean ± SD (range))	12.5 ± 9.6 (5–30)
Angiographic follow up outcome (*n* = 43)	
Stable or improved	39 (90.7)
Recurrence	4 (9.3)

^a^One patient had two aneurysms treated with LVIS stent (63 patients with 64 aneurysms) and this patient has Angiographic follow-up

### Angiographic outcomes

**Fig. 1:**
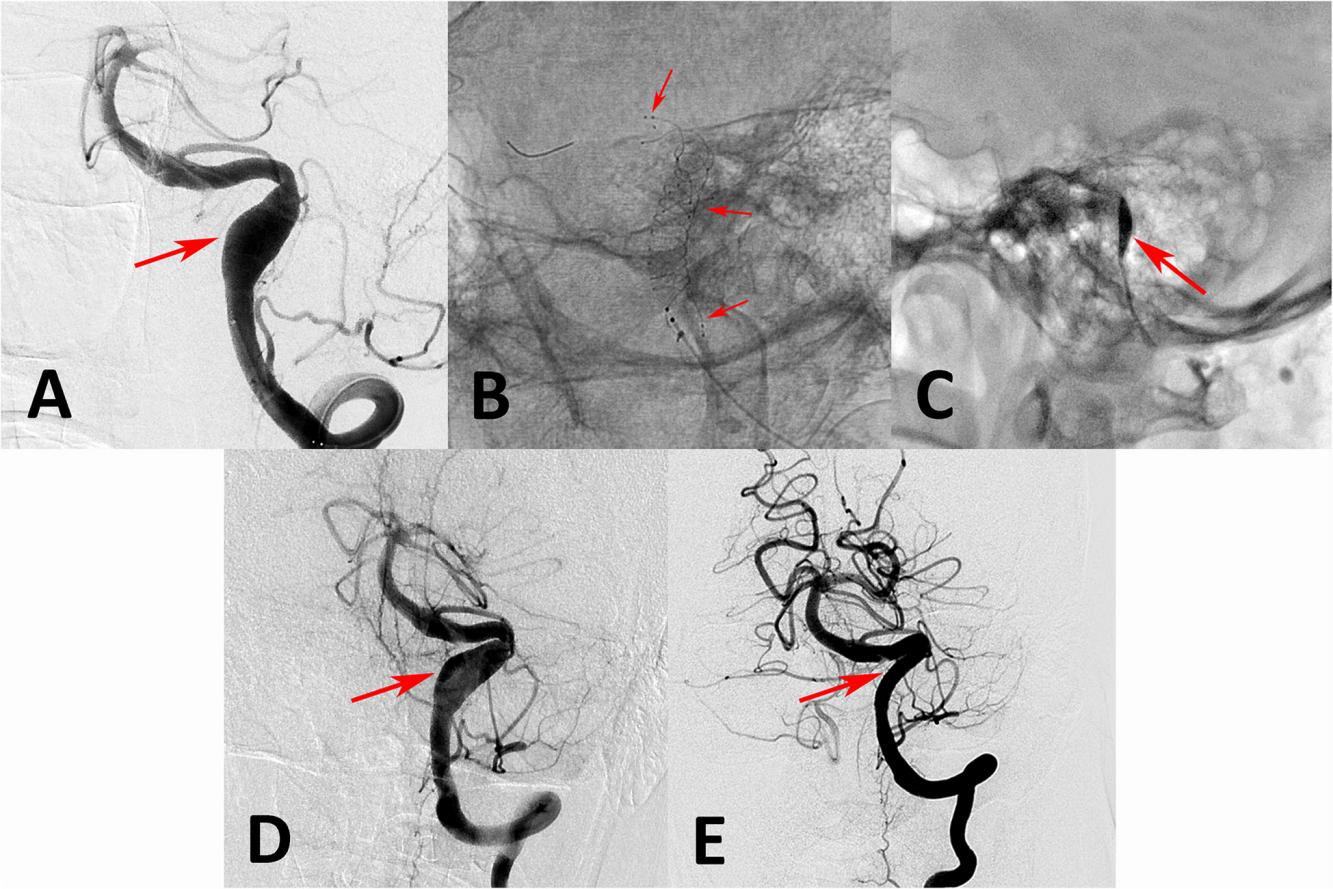
A vertebral artery aneurysm was treated with two LVIS stents without coiling. Compared with the anteroposterior position in the preoperative angiographic images (**a** arrow), after we deployed two overlapping LVIS stents (**b** arrows), the contrast residual time in the aneurysm increased (**c** arrow). However, the aneurysm was still patent in immediate post-procedure angiographic images (**d** arrow). At the 6-month follow-up angiography, the aneurysm was completely occluded (**e** arrow)

**Fig. 2:**
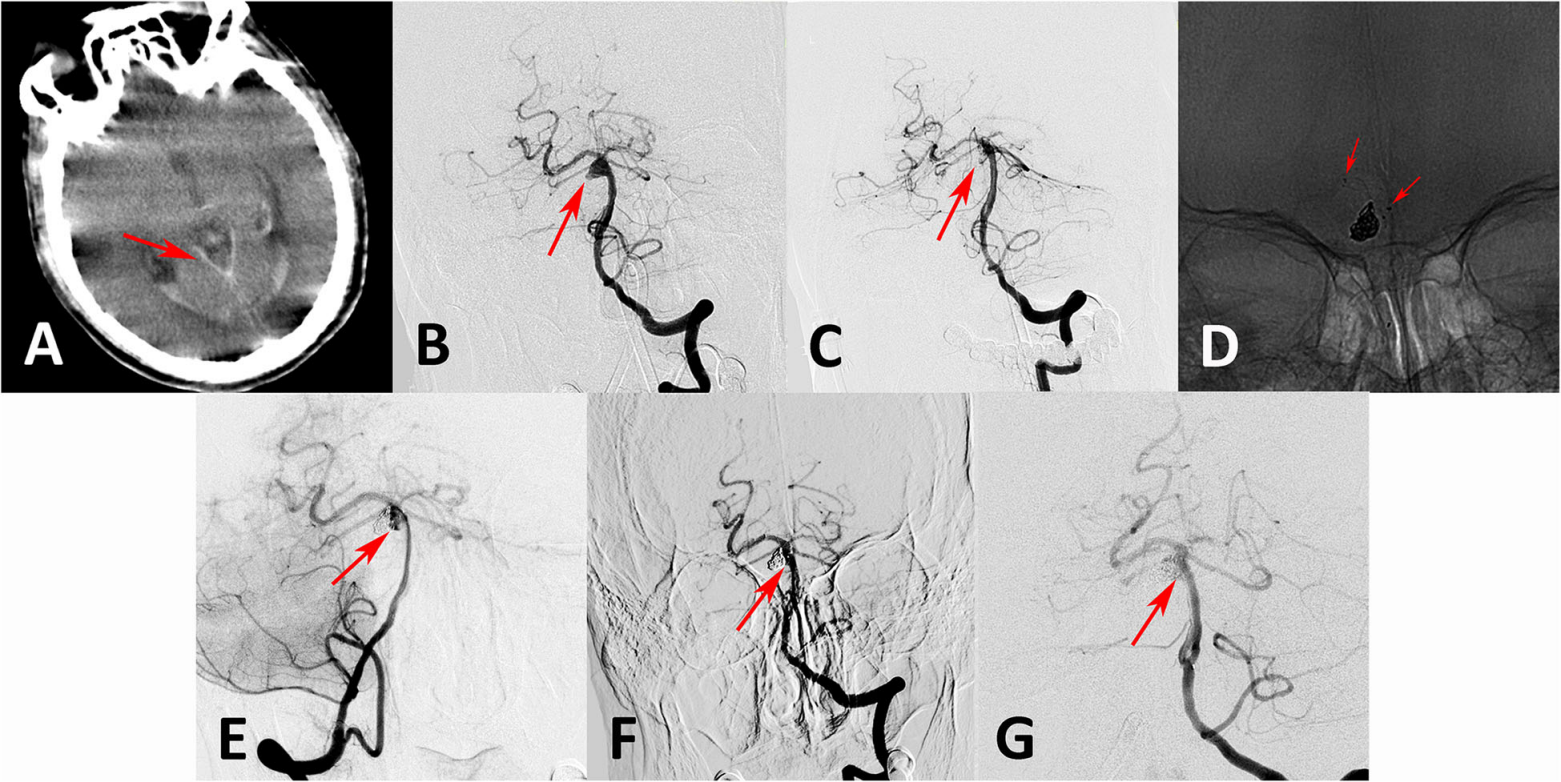
A ruptured basilar trunk aneurysm with a daughter sac was treated with LVIS-assisted coiling, and computed tomography confirmed subarachnoid hemorrhage (**a** arrow). Compared with the anteroposterior position in the preoperative angiographic images (**b** arrow), the aneurysm was completely occluded after treatment in the immediately post-procedure angiograph (**c** arrow). The LVIS stent was placed across the neck of the aneurysm followed by embolization with coils (**d**). At the 6-month follow-up angiography, the aneurysm had recanalized (**e** arrow). Repeat coiling was performed, and the residual aneurysm neck was occluded completely (**f** arrow). At the 7-month follow-up angiography, the aneurysm was stable and completely occluded (**g** arrow)

The angiographic outcomes are summarized in Table 2. Of the 64 aneurysms with adequate initial embolization, 59 patients underwent LVIS-assisted coiling (92.2%), and 5 received LVIS stents only (7.8%); 48 received a single LVIS (75.0%), and 16 received multiple LVIS stents (25.0%). In the five patients receiving only an LVIS stent, all aneurysms were patent in the immediately post-procedure angiographic images; however, the residual contrast time in the aneurysm was increased, and the parent vessel was patent after treatment. In the follow-up angiographic images, the aneurysmal characteristics improved (Fig. 1). In the aneurysms treated with LVIS-assisted coiling, 55 (93.2%) aneurysms had complete and near-complete occlusion at the end of the initial coil embolization, and 4 (6.8%) showed incomplete occlusion. After discharge, 43 patients with 44 aneurysms (67.2%) were followed with digital subtraction angiography from 5 to 30 months (mean ± standard deviation, 12.5 + 9.6 months). The follow-up images showed that 39 patients had stable or improved aneurysms (90.7%), and only 4 (9.3%) had recanalized. Three of the recanalized patients were treated with repeat coiling, and one received an additional LVIS stent. All of the recanalized patients achieved complete occlusion in the final follow-up images. Six-month follow-up images in one patient with a recanalized aneurysm treated with an LVIS stent with adjunctive coiling for a saccular basilar apex aneurysm showed that the aneurysm had recanalized. Repeat coiling was performed in this patient, and complete occlusion was observed in the final follow-up images (Fig. 2).

### Procedural complications and clinical outcomes

Intraprocedural or postprocedural complications occurred in 6 patients (9.6%): intraprocedural in 3 (4.8%) and postprocedural in 3 (4.8%). Regarding the intraprocedural complications, the LVIS stent was successfully deployed into the correct position across the entire length of the aneurysm in all patients. However, one patient developed partial coil loop protrusions, one developed in-stent thrombosis, and the stent failed to open completely in one patient. The coil loop was stable without pulsatile movement, and no additional stent placement was performed. We administered 10 μg/kg tirofiban (Grand Pharmaceutical Co Ltd., China) intra-arterially during the procedure for the patient with in-stent thrombosis, and we performed balloon angioplasty for the insufficient apposition in the patient with the incompletely opened stent, after which, the stent remained incompletely open. Two patients had in-stent stenosis (3.2%). However, the parent artery in the three patients remained patient, and none of the patients experienced neurological deficits after the procedures or during the clinical follow-up.

Post-procedural complications developed in three patients (4.8%), namely, two delayed thromboembolic events and one distal arterial hemorrhage. The two ischemic events developed immediately after the procedure, and resulted in one patient sustaining minor permanent morbidity (1.6%). This patient had a 7-mm fusiform aneurysm at the left vertebral artery that involved the posterior inferior cerebellar artery, which was treated with LVIS stent-assisted coiling. The patient suffered from mild vertigo and hoarseness after the procedure, and recovered by the last follow-up (mRS score of 0). Another complication occurred in a patient with a basilar tip saccular aneurysm who developed oculomotor nerve palsy after LVIS stent-assisted coiling. The parent artery was patent in follow-up imaging, although the oculomotor nerve palsy persisted on the right side, which affected the patient’s status at discharge (mRS score of 2). The post-procedure mortality rate was 1.6% because this patient died of brainstem hemorrhage after 1-month. Six-two patients (98.4%) had clinical outcome available at lasted follow-up, and 59 patients (95.2%) had favorable clinical outcomes (mRS score of 0–2). We further analyzed the risk factors in aneurysms with recanalization and complications, while there is no significant risk factor associated with aneurysm recanalization and complication (Supplement Table 1).

## Discussion

The LVIS device is a novel, self-expanding, braided stent that provides approximately 23% surface metal coverage. The smaller cell structure and higher metal coverage might result in better flow diversion compared with other currently available coil-assist stents. However, some factors, like perforator rich areas, should be taken into consideration, especially overlapping stents technique is used. In this study, our experience with the LVIS stent demonstrated that the technology can be safely used to treat vertebral and basilar artery aneurysms, with good clinical outcomes and follow-up occlusion rates.

### Angiographic results at follow-up

In this study, the immediate angiographic results demonstrated relatively lower rates of complete occlusion (50%); however, the progression to complete occlusion was quite satisfactory, with a high rate of 90.7% at the midterm follow-up. King et al. [10] reported the results of a literature review comparing the most widely-used intracranial stents (Neuroform and Enterprise) and found that initial and final complete occlusion was seen in 52.7 and 61.1%, respectively, of the patients treated with Neuroform stents. The final complete occlusion rate for the Enterprise stent was higher at follow-up (74.7%). In a recent prospective multicenter study of cerebral aneurysms treated with LVIS stents, the authors reported a total aneurysm occlusion rate of 91% on immediate postprocedure angiograms, and 92.4% at follow-up [11]. Cho et al. [12] reported that the complete obliteration rate of aneurysms undergoing LVIS-assisted coil embolization was 92.6% at the 6-month follow-up. However, these studies did not analyze the complete occlusion rate of vertebral and basilar artery aneurysms, separately. Wang et al. [8, 13] reported a series of patients treated with the LVIS device in vertebral and basilar artery aneurysms, with a complete occlusion rate in basilar artery aneurysms of 65% at 6.9 months of follow-up, and a complete occlusion rate in vertebral artery dissecting aneurysms of 76.7% at 8.3 months of follow-up. However, the follow-up periods might have been too short to identify a higher complete occlusion rate. In our study, the follow-up period was 12.5 months, which might explain why the complete occlusion rate at follow-up in our patients with vertebral and basilar artery aneurysms was higher than in previous studies. However, our patient cohort was relatively small, and the follow-up was still short. In addition, several studies reported that a higher rate of intra-procedural in-stent thrombosis for the LVIS stent, which ranged from 6.3–13.7% [14–16]. Gross et al. [17] reported that the Neuroform Atlas stent might have greater obliteration rates and lower in-stent stenosis in the treatment of aneurysm. Thus, stent assisted coiling with other stents (such as Neuroform Atlas stent) might remain treatment options in some posterior circulation aneurysms and given the lower metal coverage with lower in-stent stenosis rate. Further studies are needed to confirm its efficacy and safety.

Flow diversion has emerged as a treatment option for vertebral and basilar artery aneurysms, although this is currently an off-label use [18]. Flow diversion stents are mechanistically unique in that they have low porosity with obvious hemodynamic effects on aneurysms, which results in parent vessel reconstruction and intra-aneurysmal thrombosis, resolving the aneurysm. Several studies reported that flow diversion is a feasible and effective treatment for aneurysms in the vertebral and basilar artery. However, flow diversion in vertebral and basilar artery aneurysms carries a high risk of periprocedural stroke, and might be associated with high overall mortality [18–20]. Numerous studies showed that LVIS stents also have flow diverting effects to minimize aneurysmal hemodynamics [7, 21]. Wang et al. [7] quantified the flow diverting effects of the LVIS device, and compared the effect with the Pipeline device (Chestnut Medical Technologies, Menlo Park, CA) and the Enterprise stent. The authors found that the flow diverting effect of a single LVIS stent caused more flow reductions than the double-Enterprise stent but less than with a Pipeline device, and that double-LVIS stenting resulted in better flow diversion than a Pipeline device. However, the findings in that study were based on virtual models, and were not compared with clinical outcomes. Li et al. [21] selected two consecutive clinical cases to compare the hemodynamic aneurysmal changes after stent-assisted coiling between patients with the LVIS vs an Enterprise stent. The authors found that aneurysms treated with the LVIS device had greater reductions in blood flow velocity at the neck plane and within the aneurysm, and that such hemodynamic changes might explain the higher complete occlusion rate and lower recanalization rate in intracranial aneurysms treated with the LVIS device. The clinical results in our study also supported this potential mechanism. In immediate postprocedural angiography images, the complete and near-complete occlusion rate was 93.2% in patients treated with LVIS-assisted coiling, and the contrast residual time in the aneurysm was increased in all patients treated with LVIS stenting, alone. Importantly, 90.7% of the patients with follow-up images had stable or improved aneurysms.

### Complications

Aneurysms in the vertebral and basilar artery are rare but are associated with substantial morbidity and mortality. The risk of rupture in vertebral and basilar artery aneurysms is also higher compared with aneurysms in the anterior circulation, and managing vertebral and basilar artery aneurysms is particularly challenging. In the International Study on Unruptured Intracranial Aneurysms guidelines, aneurysm location in the vertebral and basilar artery was a predictor of poor outcomes with endovascular treatment [22]. The LVIS device is a novel, self-expanding stent, and limited data provide evidence supporting the use of the LVIS stent in the reconstructive treatment of vertebral and basilar artery aneurysms. In our study, the overall procedure-related complication rate was 9.6%, which is comparable to other studies evaluating LVIS stents in vertebral and basilar artery aneurysms [8, 13]. The risk of thromboembolic complications is relatively higher in vertebral and basilar artery aneurysms compared with anterior circulation aneurysms because the vertebral and basilar artery arteries have a rich collateral system, and a stent might disrupt the flow in collateral arteries. Incomplete stent expansion is a common cause of thromboembolic complications, especially when using wire-braided stents. In our study, the use of the LVIS device was feasible, with a high technical success rate of 98.4%. The stent failed to expand adequately in only one patient. Cho et al. [12] suggested that the LVIS stent might fold or twist in the tortuous anatomy and acute curve of the parent artery because of incomplete flaring of the braided closed-cell structure. In the study, the rate of thromboembolic complications following LVIS stenting was 4.8%, which is comparable to the rate of thromboembolic events of 4.9%, in a previous systematic review [23]. However, the rate of thromboembolic complications is higher with flow diverter stents (11.0%) [24]. The possible reason for the lower rate of thromboembolic complications with the LVIS stent might be that this stent had lower metal coverage. The advantages of LVIS stent include offering better visualization, improving wall apposition, and re-sheathable compared with other devices. Importantly, one patient in our study died of distal arterial hemorrhage. Previous studies have strongly suggested that excessive platelet inhibition was associated with an increased risk of hemorrhagic complications [25, 26]. However, platelet function testing was not performed in this study, and this testing might be helpful in the clinical determination of hemorrhagic risks before endovascular procedures. In our center, for the complex aneurysms located at vertebral and basilar artery (such as giant dissecting aneurysm, aneurysm involved vertebral-basilar junction, etc.), internal trapping technique was the initial treatment of choice for the lower recanalization rate. However, internal trapping might be not an appropriate treatment with several anatomic factors of the parent artery, including the involved important arterial branches, the dominance of the affected vertebral artery, and the insufficiency of the collateral blood supply. Flow diverter is an alternative treatment for the patients who were not suitable to internal trapping, for which treatment with flow diverter had a higher complete occlusion rate and better clinical outcome compared with conventional endovascular treatment [27]. However, flow diverter applied in the vertebral and basilar artery was off-label for high risk of thromboembolic events. Similarly, two or more overlapping LVIS stents might also give the high risk of thromboembolic complications. The clinicians should be cautious in the decision-making process regarding whether or when a flow diverter should be applied in the vertebral and basilar artery. Apart from the above treatments, stent-assisted coiling might be considered for the aneurysm of classic dissecting aneurysm and segmental ectasia, and had a favorable angiographic outcome [28].

This study has several limitations. First, this was a single-center retrospective study, and might include an inherent bias in patient selection. Second, the small sample size and mid-term follow-up may have influenced our findings, and a prospective study with a larger sample size is needed for validation. Third, other techniques such as internal trapping or flow diverters should be compared in future studies to evaluate the safety and efficacy of the LVIS device in vertebral and basilar artery aneurysms. Fourth, platelet function monitoring during dual-antiplatelet therapy was not standard procedure in this study, and a multicenter prospective trial may best evaluate the clinical usefulness of platelet function testing.

## Conclusions

Our study demonstrated the feasibility and safety of LVIS devices for treating vertebral and basilar artery aneurysms, and the mid-term complete occlusion rate, morbidity and mortality rates were acceptable. The LVIS stent was an acceptable safety profile and may represent a reasonable treatment option in the patients with vertebral and basilar artery aneurysms. Long-term and larger cohort studies are needed to validate our results.

## Supplementary Information

**Additional file 1: Supplemental Table 1.** Univariate analysis of the risk factors in aneurysms with recanalization and complications.

## Data Availability

All datasets generated for this study are included in the manuscript and/or the supplementary file.

## Abbreviations

LVIS: Low-profile Visualized Intraluminal Support
mRS: Modified Rankin Scale
