# Supplementary material for: Endovascular treatment of vertebral and basilar artery aneurysms with low-profile visualized intraluminal support device
Source: BMC Neurol. 2021 May 15;21:198. doi: 10.1186/s12883-021-02180-1 (PMC8122564; doi:10.1186/s12883-021-02180-1)
Supplement: Supplementary file 1 — Additional file 1: Supplemental Table 1. Univariate analysis of the risk factors in aneurysms with recanalization and complications. [file 12883_2021_2180_MOESM1_ESM.docx]

Supplemental Table 1 Univariate analysis of the risk factors in aneurysms with recanalization and complications.

| Characteristics | Non-recanalized aneurysms (N=39) | Recanalized aneurysms (N=4) | P value | Aneurysms without complication (N=57) | Aneurysms with complication (N=6) | P value |
| --- | --- | --- | --- | --- | --- | --- |
| Mean age (years) (mean ± SD) | 52.10 ± 11.85 | 54.00 ± 6.16 | 0.967 | 51.93 ± 11.18 | 54.50 ± 7.87 | 0.645 |
| Female, % | 11 (28.2) | 1 (25.0) | 0.892 | 17 (29.3) | 2 (33.3) | 0.837 |
| Symptoms, % | 30 (76.9) | 3 (75.0) | 0.931 | 21 (36.2) | 0 (0.0) | 0.072 |
| Smoking, % | 12 (30.8) | 3 (75.0) | 0.077 | 24 (41.4) | 1 (16.7) | 0.238 |
| DM, % | 3 (7.7) | 1 (25.0) | 0.256 | 5 (8.6) | 0 (0.0) | 0.454 |
| Hyperlipidemia, % | 4 (10.3) | 0 (0.0) | 0.501 | 5 (8.6) | 1 (16.7) | 0.520 |
| HBP, % | 20 (51.3) | 1 (25.0) | 0.317 | 31 (54.3) | 3 (50.0) | 0.872 |
| Pre-operative mRS (mean ± SD) | 0.72 ± 0.79 | 1.00 ± 0.82 | 0.395 | 0.85 ± 1.02 | 1.17 ± 0.41 | 0.120 |
| Dissecting aneurysm, % | 36 (92.3) | 3 (75.0) | 0.256 | 52 (89.7) | 5 (83.3) | 0.637 |
| Prior SAH, % | 7 (17.9) | 1 (25.0) | 0.730 | 13 (22.4) | 1 (16.7) | 0.746 |
| BA aneurysm, % | 9 (23.1) | 2 (50.0) | 0.240 | 16 (27.6) | 2 (33.3) | 0.766 |
| Aneurysm length (mm) (mean ± SD) | 9.85 ± 5.38 | 22.25 ± 15.99 | 0.076 | 10.98 ± 7.34 | 12.70 ± 8.37 | 0.573 |
| Aneurysm diameter(mm) (mean ± SD) | 7.62 ± 4.21 | 11.91 ± 5.91 | 0.202 | 7.67 ± 3.91 | 10.16 ± 7.43 | 0.730 |
| SAC, % | 34 (87.2) | 4 (100.0) | 0.446 | 4 (6.9) | 1 (16.7) | 0.396 |
| Multiple stents, % | 7 (17.9) | 2 (50.0) | 0.133 | 15 (25.9) | 1 (16.7) | 0.620 |
| Initial occlusion class |  |  | 0.076 |  |  | 0.752 |
| RS 1 | 21 (53.9) | 0 (0.0) |  | 31 (53.4) | 3 (50.0) |  |
| RS 2 | 16 (41.0) | 3 (75.0) |  | 23 (39.7) | 3 (50.0) |  |
| RS 3 | 2 (5.1) | 1 (25.0) |  | 4 (6.9) | 0 (0.0) |  |
| Follow-up duration (months) (mean± SD (range)) | 7.97 ± 4.19 | 5.25 ± 2.50 | 0.138 | 7.54 ± 3.41 | 9.40 ± 8.30 | 0.768 |

DM, diabetes mellitus; mRS, modified Rankin Scale; SAH, subarachnoid hemorrhage; BA, basilar artery; SAC, stent assisted coiling; RS, Raymond Scale;
